# Thyroid hormone protects human lung epithelial cells from cold preservation and warm reperfusion-induced injury

**DOI:** 10.1186/s12967-024-05024-x

**Published:** 2024-03-01

**Authors:** Dejan Bojic, Tanroop Aujla, Junichi Sugihara, Aaron Wong, Shaf Keshavjee, Mingyao Liu

**Affiliations:** 1 Latner Thoracic Surgery Research Laboratories, Toronto General Hospital Research Institute, University Health Network, Toronto, ON Canada; 2 Institute of Medical Science, Temerty Faculty of Medicine, University of Toronto, Toronto, ON Canada; 3 Department of Physiology, Temerty Faculty of Medicine, University of Toronto, Toronto, ON Canada; 4 Department of Surgery, Temerty Faculty of Medicine, University of Toronto, Toronto, ON Canada

**Keywords:** Static cold storage, Donor lung preservation, Bioinformatics, Mitochondrial function, Inflammatory response

## Abstract

**Background:**

Cellular stress associated with static-cold storage (SCS) and warm reperfusion of donor lungs can contribute to ischemia–reperfusion (IR) injury during transplantation. Adding cytoprotective agents to the preservation solution may be conducive to reducing graft deterioration and improving post-transplant outcomes.

**Methods:**

SCS and warm reperfusion were simulated in human lung epithelial cells (BEAS-2B) by exposing cells to low potassium dextran glucose solution at 4 °C for different periods and then switching back to serum-containing culture medium at 37 °C. Transcriptomic analysis was used to explore potential cytoprotective agents. Based on its results, cell viability, caspase activity, cell morphology, mitochondrial function, and inflammatory gene expression were examined under simulated IR conditions with or without thyroid hormones (THs).

**Results:**

After 18 h SCS followed by 2 h warm reperfusion, genes related to inflammation and cell death were upregulated, and genes related to protein synthesis and metabolism were downregulated in BEAS-2B cells, which closely mirrored gene profiles found in thyroid glands of mice with congenital hypothyroidism. The addition of THs (T3 or T4) to the preservation solution increases cell viability, inhibits activation of caspase 3, 8 and 9, preserves cell morphology, enhances mitochondrial membrane potential, reduces mitochondrial superoxide production, and suppresses inflammatory gene expression.

**Conclusion:**

Adding THs to lung preservation solutions may protect lung cells during SCS by promoting mitochondrial function, reducing apoptosis, and inhibiting pro-inflammatory pathways. Further in vivo testing is warranted to determine the potential clinical application of adding THs as therapeutics in lung preservation solutions.

**Supplementary Information:**

The online version contains supplementary material available at 10.1186/s12967-024-05024-x.

## Background

The only curative treatment option for patients with end-stage lung disease is transplantation. However, the accessibility of this life-saving procedure is bottlenecked by both the supply and availability of viable donor organs. Thus, improvements to graft preservation to expand the donor pool of transplantable organs are of great clinical interest.

The current standard practice for organ preservation is referred to as static-cold storage (SCS), a technique that involves cooling the graft to depress cellular metabolic activity and waste product accumulation. In the lungs, this involves flushing the pulmonary vasculature with a cooled acellular solution. Once washed, the lungs are inflated with a 50% oxygen gas mixture and then submerged in the cooled preservation solution at 4 °C before transplantation [1]. Although this technique is effective at maintaining lung graft viability for approximately 6–8 h, prolonged exposure to cold ischemia eventually leads to irreversible organ damage. Additionally, the graft is subjected to further injury upon the restoration of oxygenated blood flow at body temperature after revascularization. This ischemia–reperfusion (IR) induced acute lung injury is a major determinant of primary graft dysfunction and contributes to the development of chronic lung allograft dysfunction [2, 3].

Since IR is an inevitable consequence of the transplantation procedure, methods to improve cellular tolerance to this biphasic injury cascade are active areas of research in transplantation medicine. The composition of the preservation solution partially dictates the graft environment during SCS. Thus, improvements to the preservation solution formula may represent a promising window for therapeutic intervention. Our research group introduced the first preservation solution specifically tailored for clinical lung SCS, low potassium dextran solution, in 1989 [4]. Since its clinical adoption as Perfadex® solution, several studies have shown that its overall efficacy can be improved with the inclusion of additional buffering components and cytoprotective agents [5–7].

Developing a new organ preservation solution or modifying existing solutions is a time-consuming process with high cost, especially when animal models are used [8]. As such, cell culture models have been developed to simulate SCS and subsequent IR injury for the lung and other organs [9–11]. This approach allows for the efficient screening of multiple formulas or modifications, while also uncovering potential underlying molecular mechanisms. Using a human lung epithelial cell culture model, it has been found that adding cytoprotective agents (e.g., alpha 1 antitrypsin (A1AT), raffinose, glutathione, prostaglandin E1) to the Perfadex® solution, or using a nutrient-rich solution during SCS, protects donor lungs from IR-induced lung injury [12]. These observations prompted us to use this same in vitro model to explore the mechanisms of IR-induced injury further and develop new strategies to improve lung preservation solutions.

A recent transcriptomic study in human lung transplant tissue during IR revealed the activation of inflammatory and cell death related genes, accompanied by concurrent inhibition of metabolism and protein synthesis related genes [13]. This prompted us to examine if similar changes are present in the transcriptomic profiles of human lung epithelial cells following cold exposure and warm reperfusion in a cell culture model [14]. Findings from our transcriptomics data were used to identify therapeutics that may circumvent the multifactorial nature of IR injury by targeting several dysregulated cellular pathways. Herein, we investigated whether the supplementation of thyroid hormones (THs) into the conventional lung preservation solution, Perfadex®, could have measurable benefits to lung epithelial cells during simulated IR-induced injury.

## Methods

### In vitro ischemia–reperfusion lung transplantation model

Human lung epithelial cells transformed with Ad12-SV40 2B (BEAS-2B) is a non-cancer cell line, and have been previously used in lung transplant cell culture models [15–17]. Once cells reached the desired confluence, warm growth media (DMEM + 10% FBS) was replaced with cold Perfadex® solution (Vitrolife, Englewood, CO) or with cold Perfadex® solution containing different concentrations of triiodothyronine (T3, T6397, Sigma Aldrich, St. Louis, MI) or thyroxine (T4, Sigma Aldrich, T1775). Cells were stored at 4 °C in a sealed chamber filled with 50% oxygen for 6 or 18 h. Reperfusion was simulated by replacing cold solutions with pre-warmed growth media (DMEM + 10% FBS). Cells were then incubated at 37 °C with 5% CO_2_ for 2 or 4 h [15–17].

### Gene expression analysis

Gene expression analysis was conducted using a database as previously published, and detailed informatic methods have been described [14]. Original data can be found in the Gene Expression Omnibus database (GSE172222). Gene set enrichment analysis was subsequently completed by ranking genes using a previously referenced formula [13]. Clusters with four or more gene sets were chosen for detailed analysis in Cytoscape software (www.cytoscape.org) using EnrichmentMap and Autoannoate applications.

### Cellular viability

BEAS-2B cells were plated in a 96-well black wall/clear bottom microplate (ThermoFisher, 165305) at a seeding density of 2 × 10^4^ cells per well. Cells were incubated in growth media (DMEM + 10% FBS) overnight at 37 °C with 5% CO_2_. Upon reaching 60–70% confluence, the culture medium was removed, and cells were subjected to IR conditions as outlined above. After simulated reperfusion, cells were washed with warm PBS, and then 100 μL of 2 μM Calcein AM/HBSS (Hank’s balanced salt solution) was added to each well. Cells were incubated for 1 h at 37 °C with 5% CO_2_ in dark conditions. Fluorescence was measured with a Cytation microplate reader (BioTek, Winooski, VT) with the top reading mode at an excitation wavelength of 485 nm and emission of 530 nm.

### Caspase activity assay

BEAS-2B cells were plated as described in *Cellular Viability*. Caspase 3, 8 and 9 Multiplex Activity Assay Kit (ab219915, Abcam, Cambridge, UK) reagents were thawed at room temperature, and 50 μL of each caspase substrate was added to 10 mL of assay buffer and mixed well. Once cells underwent simulated IR, the culture medium from simulated reperfusion was removed. Cells were washed with PBS, and 100 μL of caspase assay solution was added to each well. Cells were incubated at room temperature and protected from light exposure for 1 h. Fluorescence was then measured using the Cytation microplate reader (BioTek) with a top reading mode at wavelengths corresponding to each caspase assay solution (Caspase 3: Ex/Em = 535 nm/620 nm, Caspase 8: Ex/Em = 490 nm/525 nm, and Caspase 9 Ex/Em = 370 nm/450 nm).

### Mitochondrial membrane potential measurement

BEAS-2B cell plating was performed as described in *Cellular Viability*, and IR was simulated as described above. Tetramethylrhodamine ethyl ester perchlorate (TMRE) Mitochondrial Membrane Potential Assay Kit (ab113852, Abcam) was used as outlined by the manufacturer guidelines. Briefly, all experimental groups were paired to have a corresponding carbonyl cyanide-4 (trifluoromethoxy) phenylhydrazone (FCCP) positive group, which had 20 μM of FCCP solution added to the well 10 min prior to the introduction of working TMRE solution. Cells were incubated in the dark at 37 °C with 5% CO_2_ for 30 minutes. After removal of the working TMRE solution, cells were washed twice with PBS and then analyzed with a Cytation microplate reader (BioTek) at excitation and emissions wavelengths corresponding to 549 nm/575 nm, respectively.

### Mitochondrial superoxide production assay

BEAS-2B cell plating was performed as described in the * Cellular Viability,* and IR was simulated as described above. Mitochondrial Superoxide Detection Kit (Fluorometric) (Abcam, ab219943) reagents were thawed at room temperature and protected from light in preparation. MitoROS 580 Stain working solution was created by adding 17.5 μL of 500X MitoROS 580 stain stock solution to 7 mL of assay buffer. Immediately following simulated reperfusion, growth media was removed, and cells were washed with 50 μL of assay buffer. Assay buffer was replaced with 100 μL of MitoROS 580 stain working solution. Cells were incubated at 37 °C with 5% CO_2_ for 1 h. Fluorescence was then measured using the Cytation microplate reader (BioTek) with the bottom read mode at excitation and emissions wavelengths corresponding to 540 nm/590 nm, respectively.

### NanoString

NanoString analysis was performed to determine the effects of T3 or T4 treatment on simulated IR-induced gene expression. RNA extraction, purification and quality control were conducted as described above. nCounter Human Organ Transplant Panel (NanoString Technologies, Seattle, WA) was used, which includes 758 genes covering the core pathways and processes of the host response to transplanted tissue and 12 internal reference genes for data normalization. ABCF1 was used in this experiment to normalize target genes. Gene set enrichment analysis was used by ranking genes, and clusters with four or more gene sets were chosen for detailed analysis as described above [13].

### Statistics

Statistical analyses were performed using GraphPad Prism 9.0 (GraphPad Software, San Diego, CA). One-way ANOVA was used to compare multiple groups, followed by Tukey post-hoc test. Unless stated otherwise, values are presented as mean ± SD, and *p* values < 0.05 are considered statistically significant. Significance is designated as * p < 0.05, ** p < 0.01, and *** p < 0.001.

## Results

### Warm reperfusion after prolonged cold preservation upregulates expression of genes related to inflammation and cell death in human lung epithelial cells

**Fig. 1:**
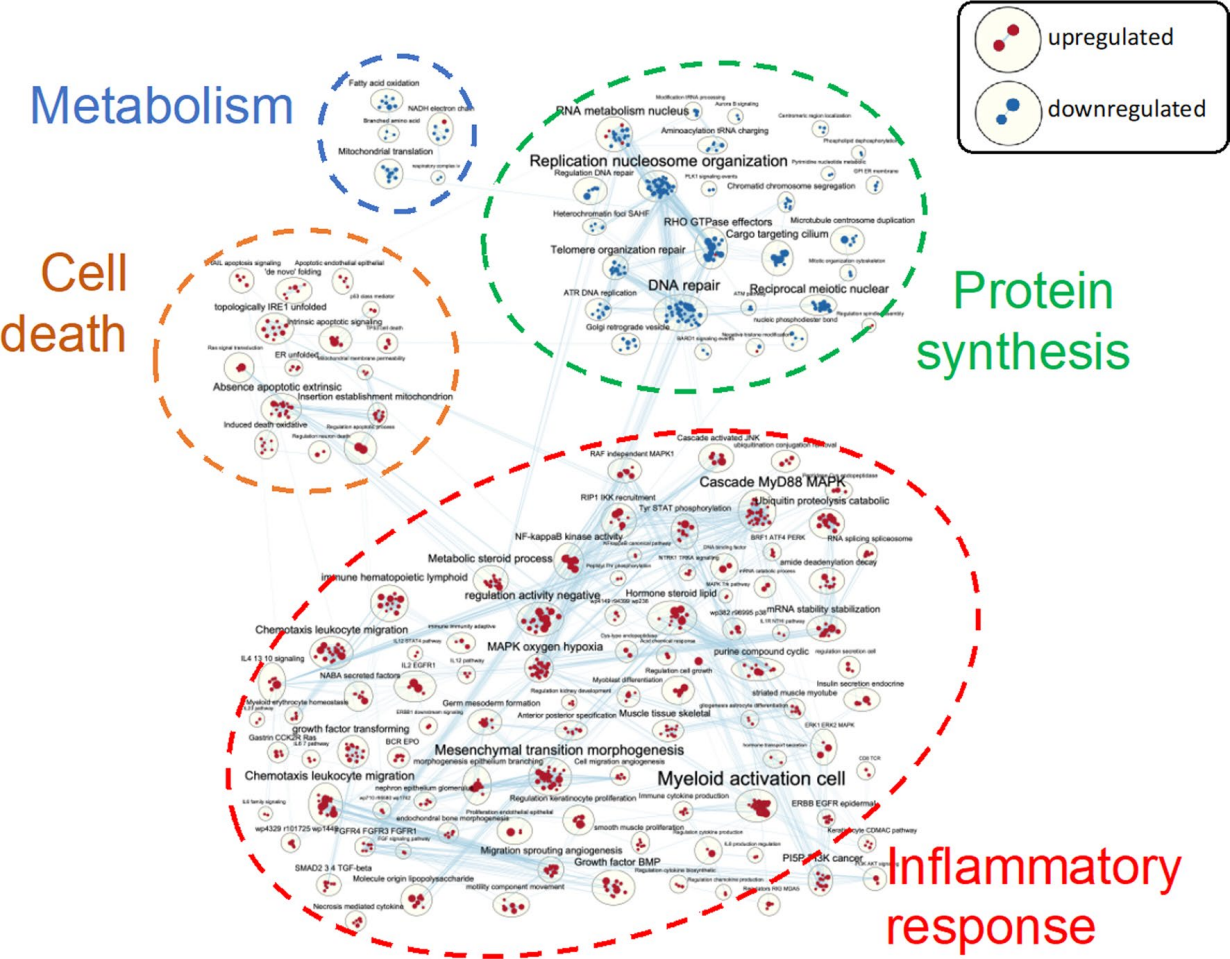
Prolonged cold preservation (18 h) and warm reperfusion (2 h) activates genes related to inflammation and cell death, and downregulates genes related to protein synthesis and metabolism in human lung epithelial cells (BEAS-2B)

To determine the effects of IR conditions on gene expression profiles in human lung epithelial cells, BEAS-2B cells were exposed to SCS, and reperfusion was simulated with pre-warmed serum-containing culture medium (Additional file 1: Fig. S1A). Total RNA was extracted and subjected to transcriptomic studies, as previously described [14]. After cells were exposed to 6 h of SCS in Perfadex® solution (a condition clinically used for donor lung preservation), 2 h of warm reperfusion induced enrichment in several gene clusters with an up-regulation of inflammation and down-regulation of protein synthesis (Additional file 1: Fig. S1B). When SCS was extended to 18 h (a condition that can induce IR injury in vitro), 2 h of warm reperfusion further up-regulated multiple gene sets associated with inflammation and cell death, and down-regulated gene sets related to metabolism and protein synthesis (Fig. 1). These results are very similar to those described from a bioinformatic study on human lung transplant tissue [13]. Our findings suggest that prolonged SCS followed by warm reperfusion in human epithelial cells is a good model to explore the mechanisms of IR injury and potential therapeutics.

### Adding T3 or T4 to the preservation solution enhances cell viability and inhibits caspase activation in human epithelial cells

A recent bioinformatics study showed that in the thyroid tissue of mice with congenital hypothyroidism, there is a downregulation of gene clusters related to cellular metabolism, followed by an up-regulation of genes related to inflammation [18]. THs are critical for many essential cellular functions, especially metabolism [19] and protein synthesis [20]. THs are also involved in cell growth, differentiation [21], and in preventing serum-starvation-induced cell death [22]. Moreover, THs are modulators of immune activities at the cellular level [23]. These important functions prompted us to evaluate whether adding THs to the preservation solution during SCS could improve cell viability, morphology, mitochondrial function, and inflammatory responses following warm reperfusion.

**Fig. 2:**
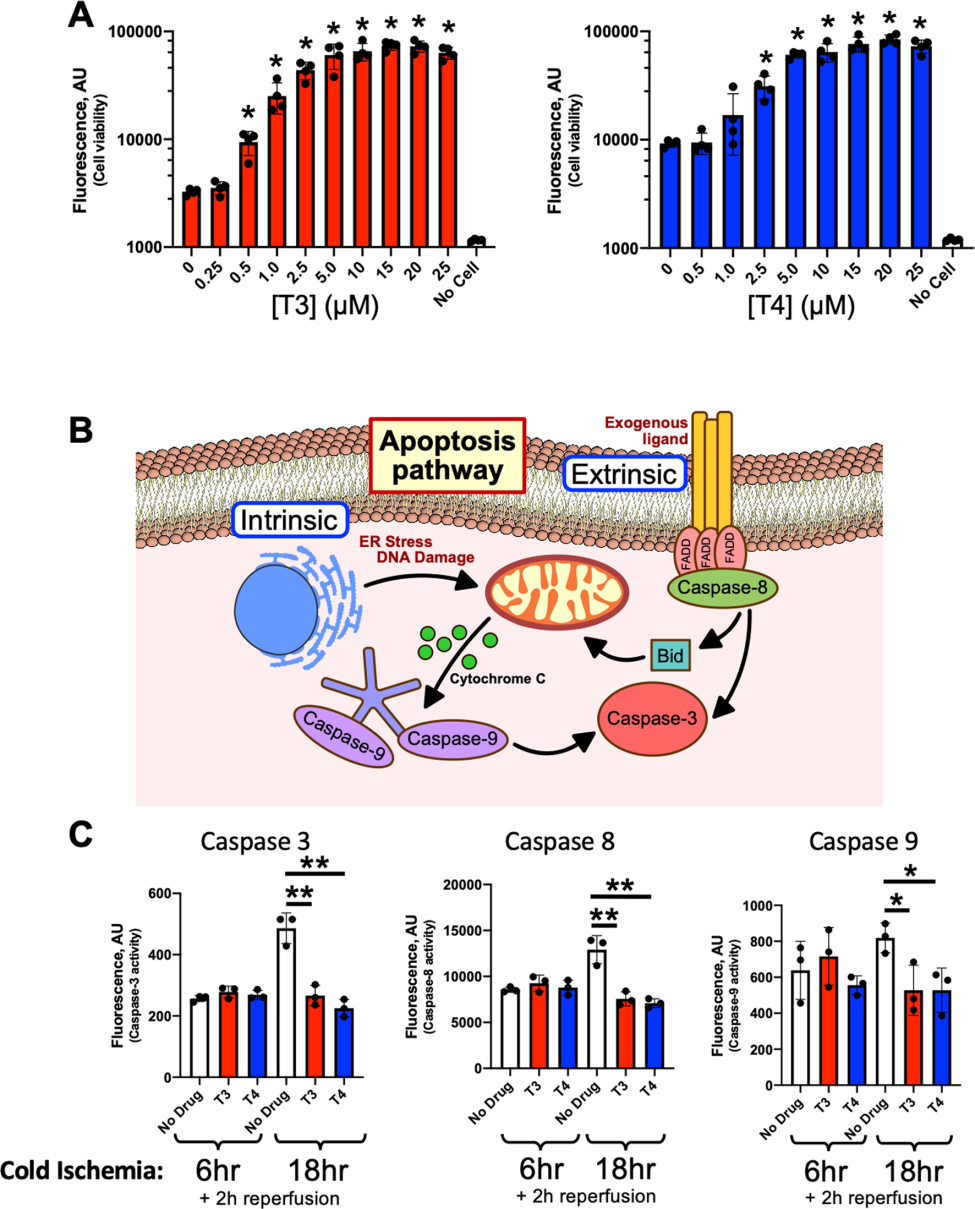
Adding TH to a cold preservation solution protects cell viability and inhibits caspase activation during warm reperfusion. **A** T3 or T4 dose-dependently improved cell viability after 18 h cold preservation and 4 h reperfusion (*p < 0.05 compared with no treatment control). **B** Role of caspase 3, 8, and 9 in mediating apoptosis. **C** T3 (2.5 μM) or T4 (25 μM) treatment prevented activation of caspase 3, 8, and 9 after 18 h CIT and 2 h reperfusion. N = 4 samples/group, one-way ANOVA followed by Tukey post-hoc test. *p < 0.05; **p < 0.01

Thyroxine (T4) is the major TH released from the thyroid into circulation; however, T4 can be converted to a more bioactive form, Triiodothyronine (T3), via deiodinases. Increasing concentrations (0.25–25 μM) of either T3 or T4 were added to the Perfadex® solution during 18 h SCS, which was followed by 4 h of simulated warm reperfusion. Cell viability was evaluated with a Calcein AM assay. T3 or T4 treatments resulted in a dose-dependent increase in cellular viability (Fig. 2A). Based on these results, T3 and T4 concentrations for subsequent studies were kept at 2.5 μM and 25 μM, respectively.

Since cells were preserved at low temperatures and warm reperfusion was only 4 h, we postulated that increased cellular viability was related to a suppression of cell death following TH treatment. Apoptosis can be induced by both extrinsic and intrinsic pathways, initiated by caspase 8 and caspase 9, respectively. Eventually, caspases 8 and 9 activate executioner caspases (mainly caspase 3) which leads to cell death (Fig. 2B). Following 6 h SCS and 2 h warm reperfusion, activity levels of all three caspases in the T3 and T4 treated groups did not significantly differ from the untreated condition (Fig. 2C). However, when SCS was extended to 18 h (the time at which in vitro cellular injury is more pronounced), untreated cells had significantly increased caspase activity after warm reperfusion relative to their paired 6 h condition. Additionally, T3 or T4 exposed cells had significantly decreased activities of all 3 measured caspases (caspase 3, 8, and 9) compared to untreated cells (Fig. 2C). These findings indicate that the increased cell viability after TH administration is partially due to reduced apoptosis.

### Preservation solution supplemented with T3 or T4 improves cellular morphology and mitochondrial function

**Fig. 3:**
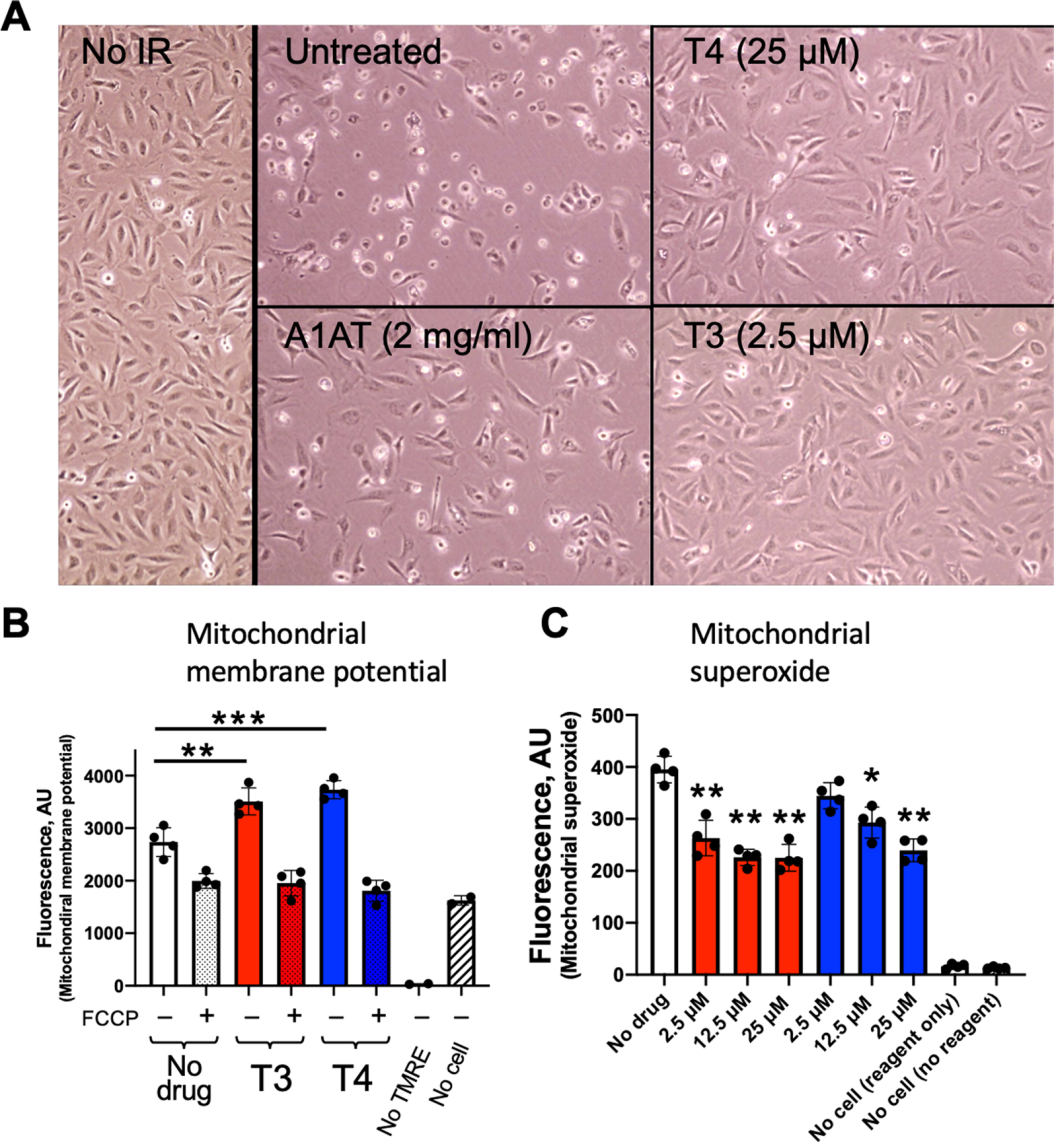
Adding TH to a cold preservation solution prevents cell injury during warm reperfusion. **A** Cell morphology was assessed with phase contrast microscopy. T3 (2.5 µM) or T4 (25 µM) was added to Perfadex® solution during cold preservation for 18 h, followed by warm reperfusion for 2 h. Perfadex® solution alone or with A1AT (2 mg/mL) was used for comparison. **B** T3 (2.5 μM) or T4 (25 μM) treatment elevated mitochondrial membrane potential. The specificity of the test was verified with FCCP treatment, which is an uncoupler of oxidative phosphorylation. **C** T3 or T4 treatment reduced mitochondrial superoxide production dose-dependently. N = 4 samples/group, one-way ANOVA followed by Tukey post-hoc test. *p < 0.05; **p < 0.01; ***p < 0.001, compared to no drug control

Cell morphology was examined with phase contrast microscopy. After 18 h of SCS, 2 h warm reperfusion-induced apparent changes in cell morphology with alterations in cell shape and decreased cell attachment (Fig. 3A, untreated). In our previous studies, A1AT treatment during SCS showed protective effects on cell morphology [11, 12]; therefore, A1AT was used as a positive control group in the present study. Cellular morphology and density in T3 (2.5 μM) and T4 (25 μM) treated groups were comparable to that of non-IR control and A1AT (2 mg/mL) treated groups (Fig. 3A).

The mitochondria play a fundamental role in several essential cell functions, such as promoting cellular metabolism [24]. Further, mitochondrial dysfunction has been linked to an increased risk of primary graft dysfunction in the lung transplant setting [25]. As such, we evaluated mitochondrial function in BEAS-2B cells exposed to T3 (2.5 μM) or T4 (25 μM) supplemented Perfadex® solutions. T3 or T4 treated cells demonstrated significantly higher mitochondrial membrane potential after 2 h of warm reperfusion, compared to no drug-treated controls (Fig. 3B). Further, treatment of cells with increasing concentrations (2.5 μM to 25 μM) of either T3 or T4, significantly decreased mitochondrial superoxide production in a dose dependent manner relative to the untreated controls (Fig. 3C).

### Adding T3 or T4 to the preservation solution prevented IR-induced expression of inflammatory genes

**Fig. 4:**
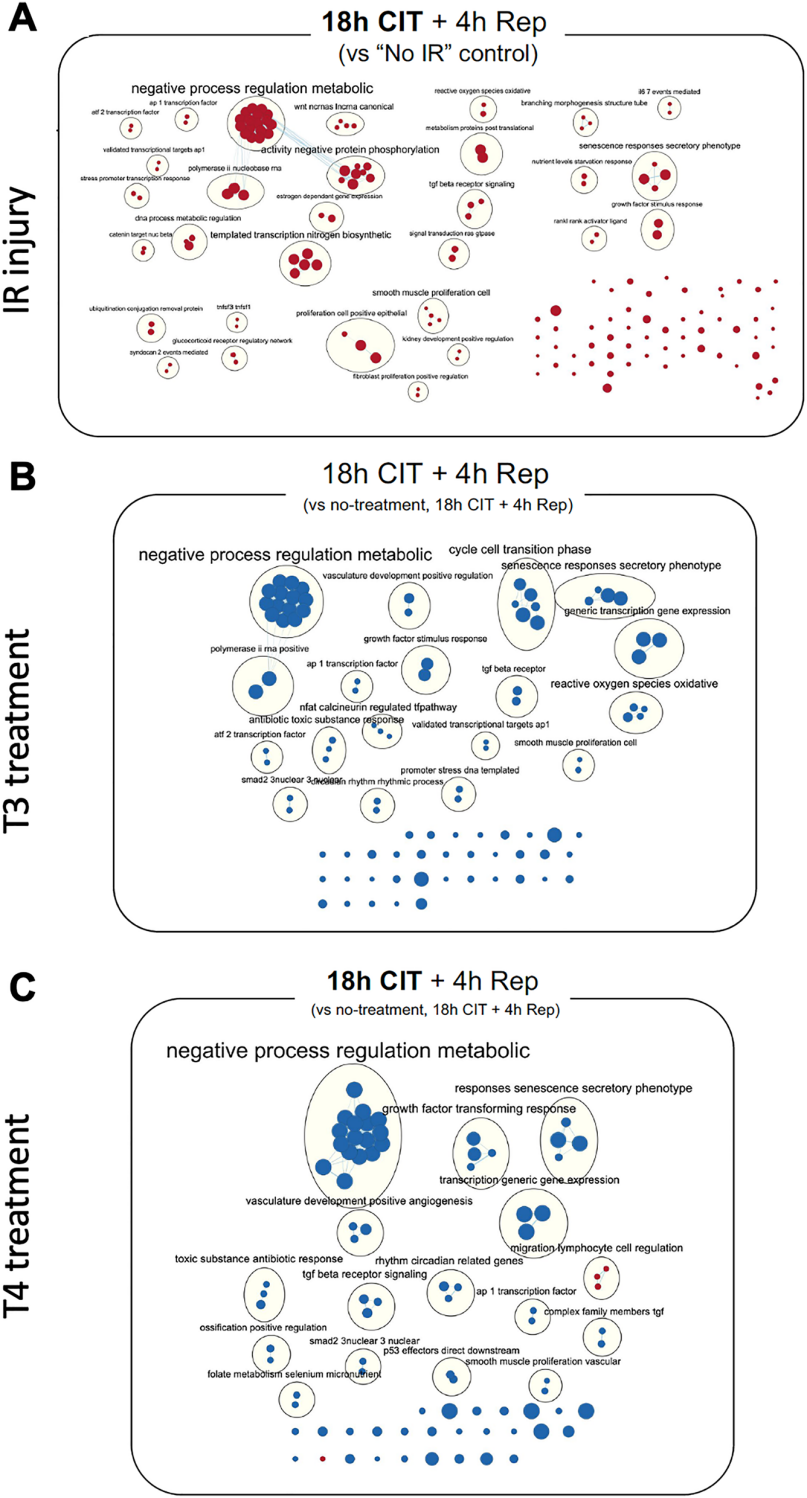
Adding TH to a cold preservation solution prevents prolonged cold preservation and warm reperfusion-induced expression of genes related to organ transplantation. Cells were treated with or without T3 (2.5 μM) or T4 (25 μM) during 18 h cold preservation, followed by 4 h warm reperfusion. Gene expression was assessed with NanoString. The pathways of gene expression were assessed with Gene Set Enrichment Analysis. **A** Compared to the control group, IR activated multiple gene pathways related to human organ transplantation. **B**, **C** T3 or T4 treatment downregulated human organ transplantation pathways

To determine the effects of THs on IR-induced gene expression, we used NanoString to study a well-defined panel of genes related to human organ transplantation. Differentially expressed genes were then evaluated with GSEA. Cells were preserved at 4 °C for either 6 h or 18 h in SCS conditions, followed by 4 h of warm reperfusion (Additional file 1: Fig. S2A). T3 (2.5 μM) or T4 (25 μM) was added to Perfadex® solution during SCS. Relative to normally cultured cells, cells exposed to 6 h SCS and 4 h reperfusion had gene enrichment in interferon signalling and virus defense response (Additional file 1: Fig. S2B). When T3 and T4 were introduced during the 6 h SCS stage, the gene expression signature without TH supplementation was eliminated (Additional file 1: Fig. S2C) and replaced by alternate gene pathways as seen specifically with T4 supplementation (Additional file 1: Fig. S2D). When SCS was extended to 18 h with a subsequent 4 h reperfusion, and compared to normally cultured cells, multiple genes were activated in diverse pathways ranging from reactive oxygen species oxidation to negative regulation of metabolism (Fig. 4A). Alternatively, T3 or T4 treatment countered majority of these gene pathways (Fig. 4B, C).

**Fig. 5:**
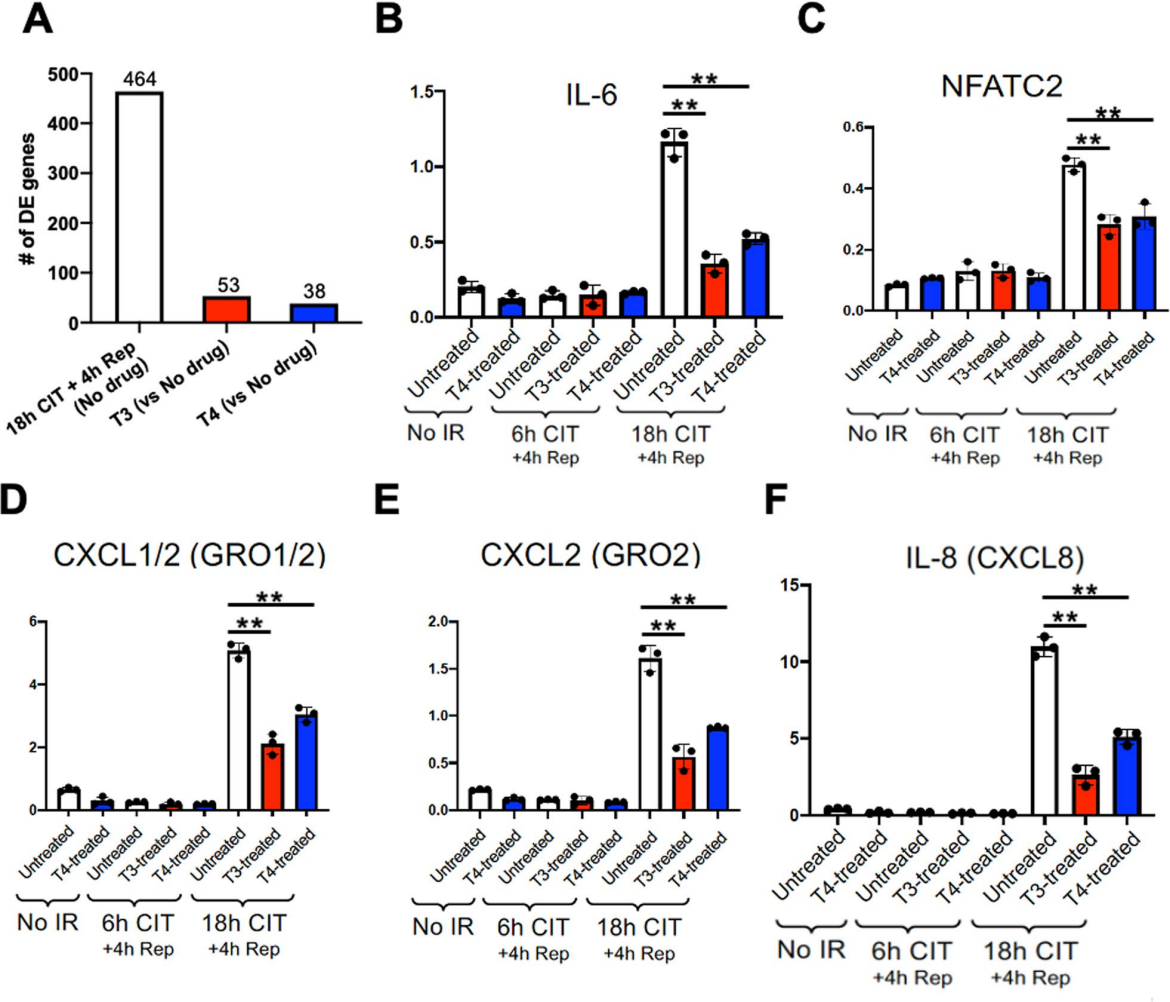
Adding TH to a cold preservation solution prevents IR-induced inflammatory gene expression. Cells were treated with or without T3 (2.5 μM) or T4 (25 μM) during 6 or 18 h cold preservation, followed by 4 h warm reperfusion. Gene expression was assessed with NanoString and expressed as a ratio relative to the housekeeping gene ABCF1. **A** T3 or T4 reduced the numbers of differentially expressed (DE) genes. **B**–**F**. T3 or T4 prevented IR-induced expression of IL-6, NFATC2, CXCL1/2, CXCL2, and IL-8 (compared to untreated group after 18 h cold preservation). N = 3 samples/group, one-way ANOVA followed by Tukey post-hoc test. **p < 0.01

Compared with cells that were not exposed to IR, those which were exposed to 18 h of SCS with 4 h reperfusion had significantly increased expression of 464 genes in the Human Organ Transplant Panel, while T3 and T4 treated groups only had 53 and 38 significantly increased genes, respectively (Fig. 5A). Additionally, cells exposed to 18 h SCS followed by 4 h reperfusion induced upregulation of IL-6 (inflammatory cytokine), NFATC2 (signal transduction protein), and CXCL1/2, CXCL2, and CXCL8 (inflammatory chemokines), which were all significantly reduced by T3 or T4 treatment (Fig. 5B–F).

## Discussion

Progressive deterioration of graft viability with prolonged preservation continues to be a significant hurdle in organ transplantation. Thus, the identification of therapeutic targets of IR injury for pharmacological intervention is a major research goal in transplantation medicine. In the present study, we found that the transcriptomic profile of human lung epithelial cells exposed to simulated transplant conditions had a signature profile like those found in clinical human lung transplants [13] and in the thyroid glands of mice with congenital hypothyroidism [18]. This prompted us to investigate whether incorporating THs into the standard clinical lung preservation solution could protect against IR injury. We found that TH intervention during the SCS period ameliorates many of the hallmarks of IR injury, such as cellular apoptosis, disruption of cellular morphology, mitochondrial dysfunction, and inflammatory gene expression.

### THs in donor preconditioning for organ transplantation

Much of the research on THs for the purposes of transplantation has involved preconditioning therapy, where THs are delivered intravascularly to identified donors prior to organ procurement. TH preconditioning for organ transplantation has been studied in the liver [26], kidney [27–29], and heart [30, 31]. Further, retrospective clinical studies have shown that TH preconditioning results in more transplantable organs originating from brain-dead organ donors [32]. However, prospective randomized studies have not shown the same benefit [31, 33, 34]. In this study, we explored adding THs directly to the lung preservation solution during SCS. This novel strategy could offer direct protection to donor organs. The potential mechanisms of THs on donor organs discovered from these preconditioning studies could be good references to interpret our results.

### The addition of THs in the preservation solution enhances cell survival

Results of the current study demonstrate that T3 or T4 treatment significantly mitigates apoptotic cell death through the inhibition of caspase 3, 8, and 9 after 18 h of SCS and warm reperfusion. In neonatal rat cardiomyocytes, THs prevented serum starvation-induced cell death through the activation of the Akt pathway [22]. In a Langendorff-perfused rat heart model, adding T3 during reperfusion improved recovery of heart function and limited apoptosis [35]. MAPK mediates inflammatory response and cell death in an IR lung transplantation model [36]. The effects of THs on Akt, MAPK and other pathways should be studied further. In the context of lung transplantation, apoptosis has been observed in human lung grafts during reperfusion [5], and apoptosis inhibitors have reduced IR injury in pre-clinical transplant models [37, 38]. The use of THs to promote cell survival during lung transplantation is a promising approach to improve organ quality and viability.

While apoptosis was the primary cell death pathway evaluated in the present study, we have previously shown a dynamic progression from apoptosis to necrosis when SCS is extended prior to reperfusion [39]. In rodent kidney IR models, TH preconditioning has been shown to reduce renal tubular necrosis [27]. While not evaluated in the present study, programmed cell death pathways, including necroptosis, pyroptosis, ferroptosis, and autophagy-related cell death, have been implicated in IR injury during lung transplantation [40]. Thus, the potential effects of THs on different types of programmed cell death in the setting of lung IR injury should be studied in the future to fully characterize its protection.

### The addition of TH in the preservation solution protected cytoskeletal structure and mitochondrial function

Maintenance of organ function is closely linked to cytoskeletal integrity. In the present study, we demonstrated that adding THs to the donor lung preservation solution during SCS partially preserved human epithelial cell morphology. This is consistent with previous reports showing that renal tubular cell ultrastructure [27] and renal and hepatic histology are preserved with TH preconditioning [28, 41]. In rodents, TH administration protected alveolar fluid clearance after ventilatory injury [42] or regulated fluid balance in intact lungs [43].

In the present study, we observed significant up-regulation of mitochondrial membrane potential following T3 or T4 treatment after simulated IR injury, which suggests that THs may help restore cellular energy deficits. While we did not measure cellular ATP levels directly, THs are important regulators of mitochondrial oxidative phosphorylation, and higher mitochondrial membrane potential may facilitate ATP production. Another observed benefit of TH supplementation was a dose-dependent reduction in mitochondrial superoxide production, which may reduce cell death and inflammatory responses. Using the same IR injury model, we have previously shown that blocking cell death related pathways also up-regulates mitochondrial membrane potential and reduces mitochondrial reactive oxygen species production, improving basic cellular function [15, 16].

### The addition of THs in the preservation solution inhibited IR-induced inflammatory responses

Transcriptomic evaluations from our in vitro work revealed genes related to inflammatory responses were significantly upregulated in a transplant cell culture model and paralleled findings previously observed in clinical samples [13]. NanoString, nCounter Human Organ Transplant Panel, is a powerful tool to determine the expression of selected genes of interest related to human organ transplant. Using this panel, we further demonstrated an upregulation of genes relevant to organ injury in untreated cells exposed to transplant conditions, which were effectively blocked by T3 or T4 treatment during SCS. The notable suppression of inflammatory cytokine genes (IL-6, NFATC2, CXCL1/2, CXCL2, and IL-8) provides strong support for the future adoption of THs into lung preservation solutions, given the immense inflammatory cascade that can arise after IR injury. Similar suppression of inflammatory signals in TH preconditioning studies has been observed in animal studies. In rat hepatic and renal IR injury models, TH preconditioning decreased IL-6 gene expression and circulation of pro-inflammatory cytokines (TNF-α, IL-6, and MIP-1α), respectively [28, 41]. Together these studies suggest the benefits of TH supplementation on inflammatory signals can be observed in vivo, and thus the administration of these hormones into preservation solutions may replicate some of the benefits reported with preconditioning studies.

### Limitations

Although the present study demonstrated the addition of THs into a lung preservation solution can mitigate IR injury in a human lung epithelial cell culture model, further in vivo testing using animal models is necessary to translate this approach towards clinical application. Upon transplantation, the preservation solution present in the vasculature of donor lungs is limited; thus, the systemic effect of THs on the recipient should be negligible. However, the safety of this strategy needs to be tested with animal models. Another limitation is the lack of in-depth mechanistic studies evaluating the underlying mechanisms of THs. Further investigation on the effects of THs on protein synthesis and metabolism is necessary. In this regard, a proteomic and metabolomic approach to supplement transcriptomic findings should be considered.

## Conclusion

In the present study, we demonstrated the benefits of TH when it is supplemented directly into the lung preservation solution. Recent studies have shown that increasing donor lung SCS temperature from 4 °C to 10 °C improved organ quality and significantly extended preservation time by protecting mitochondrial function [44]. Using nutrient-rich solutions or adding multiple cytoprotective agents in lung preservation solutions has also improved donor lung quality [12]. Thus, it is conceivable that the coupling of these preservation approaches, with THs, may work synergistically to protect mitochondrial function and further improve graft viability.

### Supplementary Information

**Additional file 1: Figure S1.** Short cold preservation and warm reperfusion activates genes related to inflammation. A) A cell culture model that simulates cold preservation and warm reperfusion in lung transplantation. B) After 6 h cold preservation (clinically used condition), gene clusters related to inflammation were enriched during warm reperfusion in human lung epithelial BEAS-2B cells, which is also associated with the down-regulation of genes related to protein synthesis. **Figure S2.** A) Experimental design. Cells were treated with or without T3 (2.5 μM) or T4 (25 μM) for 6 h or 18 h cold preservation, followed by 4 h warm reperfusion. Gene expression was assessed with NanoString for pathways associated with human organ transplantation. B) Compared with no-IR treated cells, IR-exposed cells had increased expression in interferon signalling and virus defence pathways. C and D) Reperfusion had minimum effects on differential gene expression in T3 and T4 treated cells, respectively.

## Data Availability

The datasets used and/or analysed during the current study are available from the corresponding author on reasonable request.

## Abbreviations

SCS: Static cold storage
IR: Ischemia reperfusion
A1AT: Alpha 1 Antitrypsin
TH: Thyroid hormone
TMRE: Tetramethylrhodamine ethyl ester perchlorate
FCCP: Carbonyl cyanide-4 (trifluoromethoxy) phenylhydrazone
T4: Thyroxine
DE: Differentially expressed
T3: Triiodothyronine
