# Supplementary material for: Thyroid hormone protects human lung epithelial cells from cold preservation and warm reperfusion-induced injury
Source: J Transl Med. 2024 Mar 1;22:221. doi: 10.1186/s12967-024-05024-x (PMC10908176; doi:10.1186/s12967-024-05024-x)
Supplement: Supplementary file 1 — Additional file 1: Figure S1. Short cold preservation and warm reperfusion activates genes related to inflammation. A) A cell culture model that simulates cold preservation and warm reperfusion in lung transplantation. B) After 6 h cold preservation (clinically used condition), gene clusters related to inflammation were enriched during warm reperfusion in human lung epithelial BEAS-2B cells, which is also associated with the down-regulation of genes related to protein synthesis. Figure S2. A) Experimental design. Cells were treated with or without T3 (2.5 μM) or T4 (25 μM) for 6 h or 18 h cold preservation, followed by 4 h warm reperfusion. Gene expression was assessed with NanoString for pathways associated with human organ transplantation. B) Compared with no-IR treated cells, IR-exposed cells had increased expression in interferon signalling and virus defence pathways. C and D) Reperfusion had minimum effects on differential gene expression in T3 and T4 treated cells, respectively. [file 12967_2024_5024_MOESM1_ESM.docx]

**Supplementary figure legends
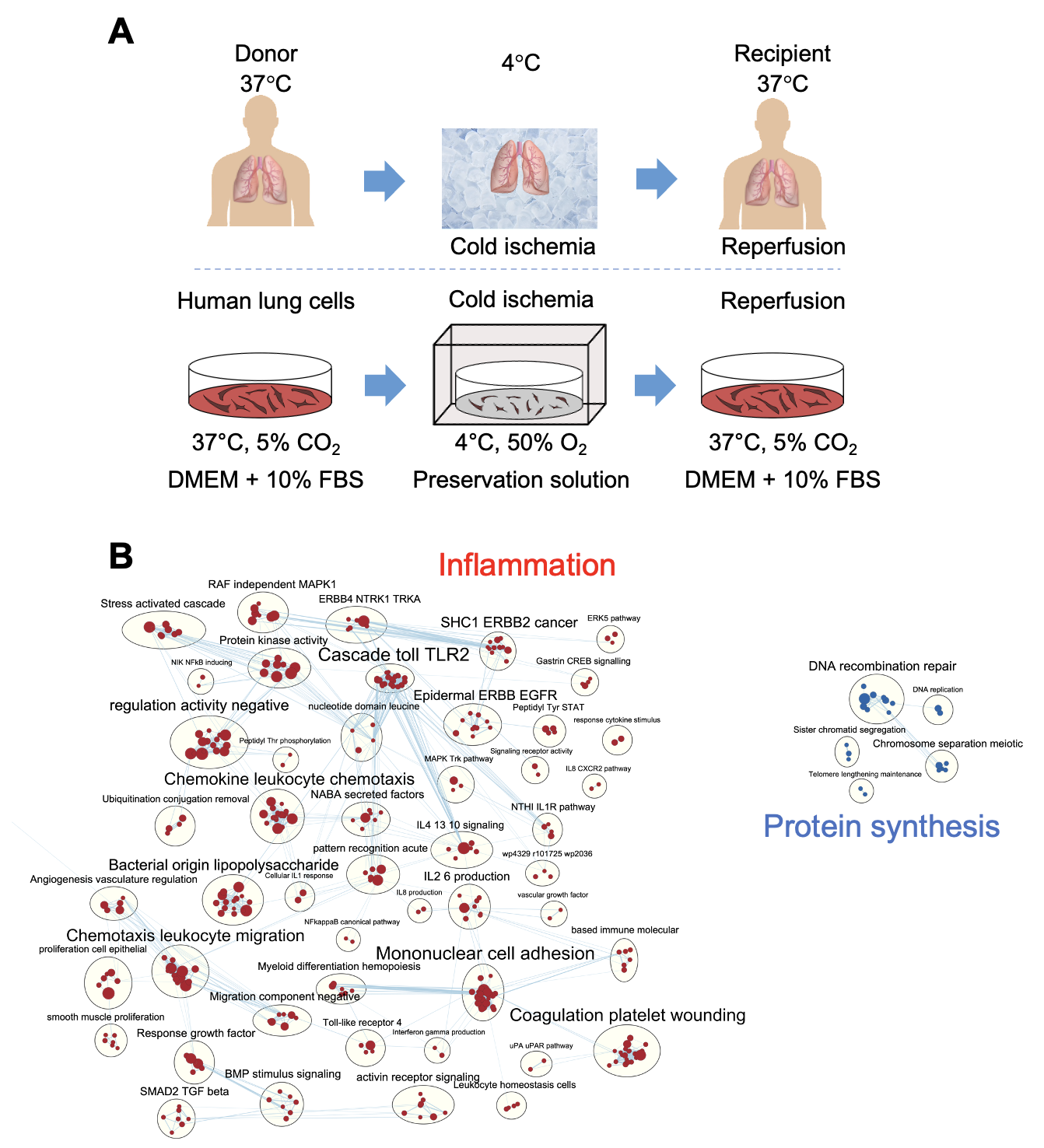
**

**Figure S1. Short cold preservation and warm reperfusion activates genes related to inflammation**. A) A cell culture model that simulates cold preservation and warm reperfusion in lung transplantation. B) After 6 h cold preservation (clinically used condition), gene clusters related to inflammation were enriched during warm reperfusion in human lung epithelial BEAS-2B cells, which is also associated with the down-regulation of genes related to protein synthesis.

**Figure S2. Effects of TH in cold preservation solution on ischemia-reperfusion (IR) induced gene expression.** A) Experimental design. Cells were treated with or without T3 (2.5 μM) or T4 (25 μM) for 6 h or 18 h cold preservation, followed by 4 h warm reperfusion. Gene expression was assessed with NanoString for pathways associated with human organ transplantation. B) Compared with no-IR treated cells, IR-exposed cells had increased expression in interferon signalling and virus defence pathways. C and D) Reperfusion had minimum effects on differential gene expression in T3 and T4 treated cells, respectively.
